# Nine anthropometric indices predict hepatic steatosis and assess liver health in adolescents: a population-based study

**DOI:** 10.3389/fped.2025.1558023

**Published:** 2025-06-09

**Authors:** Ruanchang Chen, Fangzheng Zhou, Jiayao Zhang, Huanjun Tong, Baochun Lu

**Affiliations:** ^1^ School of Medicine, Shaoxing University, Shaoxing, Zhejiang, China; ^2^ Department of Hepato-Biliary-Pancreatic Surgery, Shaoxing People’s Hospital, Shaoxing, Zhejiang, China; ^3^ School of Medicine, Zhejiang University, Hangzhou, Zhejiang, China

**Keywords:** anthropometric indices, AVI, hepatic steatosis, adolescent, NHANES

## Abstract

**Purpose:**

The increasing prevalence of obesity among adolescents has resulted in an increase in the incidence of hepatic steatosis; however, the relationship between anthropometric measurements and this condition in youth remains underexplored.

**Aims:**

To evaluate the effectiveness of nine anthropometric indicators in predicting the risk of hepatic steatosis in adolescents.

**Methods:**

We assessed several anthropometric indicators, including the abdominal volume index (AVI), body mass index, body roundness index, body adiposity index, conicity index, waist-hip ratio, waist-to-height ratio, and weight-adjusted waist index. Statistical methods such as multivariate logistic regression, smooth curve fitting, and subgroup analysis were employed. Discriminative accuracy was determined using receiver operating characteristic curve analysis, and a tool based on the optimal Youden index was developed.

**Results:**

All nine indices were significantly correlated with hepatic steatosis in adolescents. AVI demonstrated the strongest predictive ability, with an area under the curve of 0.8454 (95% confidence interval: 0.8221–0.8687, best threshold: 14.9992). Variations in predictive accuracy were observed across racial and ethnic subgroups, highlighting the importance of demographic factors.

**Conclusion:**

All nine anthropometric indices are associated with hepatic steatosis, with AVI emerging as the most effective tool for assessing liver health in adolescents.

## 1 Introduction

The rising prevalence of obesity among adolescents has become a critical global public health issue. In the United States, 20.9% of adolescents aged 12–19 are classified as obese (1). This excessive weight not only increases the risk of various health complications but also exacerbates the likelihood of experiencing physical and psychological conditions, such as depression (2), disrupted sleep patterns (3), early onset of puberty (4), high blood pressure (5), and non-alcoholic fatty liver disease (NAFLD) (6). Alarmingly, NAFLD affects 36.1% of obese adolescents (7), underscoring the significance of addressing this liver-related condition, which can progress to more severe health problems if left untreated. Severe hepatic steatosis, commonly referred to as fatty liver, results from the accumulation of excess fat in liver cells. Metabolic syndrome—defined as a cluster of metabolic abnormalities, including central obesity, insulin resistance, dyslipidemia, and hypertension—is closely associated with NAFLD. In pediatric populations, the definition of metabolic syndrome is adjusted for age and developmental stage, but its presence significantly increases the risk of hepatic steatosis and its progression (8). As obesity and NAFLD rates continue to rise among adolescents, the global incidence of hepatic steatosis is also increasing (9). This condition can impair liver function and potentially lead to more severe liver diseases, such as fibrosis, cirrhosis, and even liver cancer, which pose substantial long-term health risks (10). Early detection of hepatic abnormalities in adolescents is crucial, as prompt intervention can slow disease progression and improve long-term health outcomes.

Basic tools such as tape measures and scales allow parents to monitor their children's physical development. Various anthropometric indicators, including body mass index (BMI) (11), weight-adjusted waist index (WWI) (12), and waist-to-height ratio (WHtR) (13), have been associated with hepatic steatosis. These indices are valuable for prevention and diagnostic purposes. These metrics are essential in identifying the risk of hepatic steatosis, enabling early intervention and timely medical diagnosis. However, most research has focused on adults, and studies on adolescents remain limited and inconclusive.

The primary objective of this study is to assess and compare the effectiveness of nine distinct anthropometric indicators in predicting hepatic steatosis among adolescents. These indicators include the body shape index (ABSI), abdominal volume index (AVI), BMI, body roundness index (BRI), body adiposity index (BAI), conicity index (CI), WHtR, waist-hip ratio (WHR), and WWI. Each of these indices offers a unique approach to identifying hepatic steatosis, a condition characterized by abnormal fat accumulation in liver cells. BMI provides a general measure of body fat, ABSI assesses body shape-related health risks, AVI focuses on abdominal fat, BAI estimates adiposity, BRI gauges body roundness, CI evaluates body contour, WHR compares waist to hip size, WHtR relates waist circumference to height, and WWI adjusts waist circumference for weight. This comprehensive approach enhances our ability to detect adolescents at risk of hepatic steatosis. Data for this study were drawn from the NHANES 2017–2020 dataset. Our analysis aims to establish the most effective cutoff values for these indices, thereby refining the risk assessment for hepatic steatosis in adolescents. The results will contribute to the early detection of high-risk individuals, facilitating timely intervention and reducing the incidence of hepatic steatosis, ultimately improving the health outcomes for adolescents.

## 2 Material and methods

### 2.1 Study design and participants

Data for this study were obtained from the National Health and Nutrition Examination Survey (NHANES), a comprehensive health assessment conducted by the National Center for Health Statistics (NCHS) under the Centers for Disease Control and Prevention (CDC). The primary objectives of NHANES are to collect and evaluate dietary and health data from across the United States, monitor health trends, and provide scientific evidence to inform public health policies (14).

**Figure 1:**
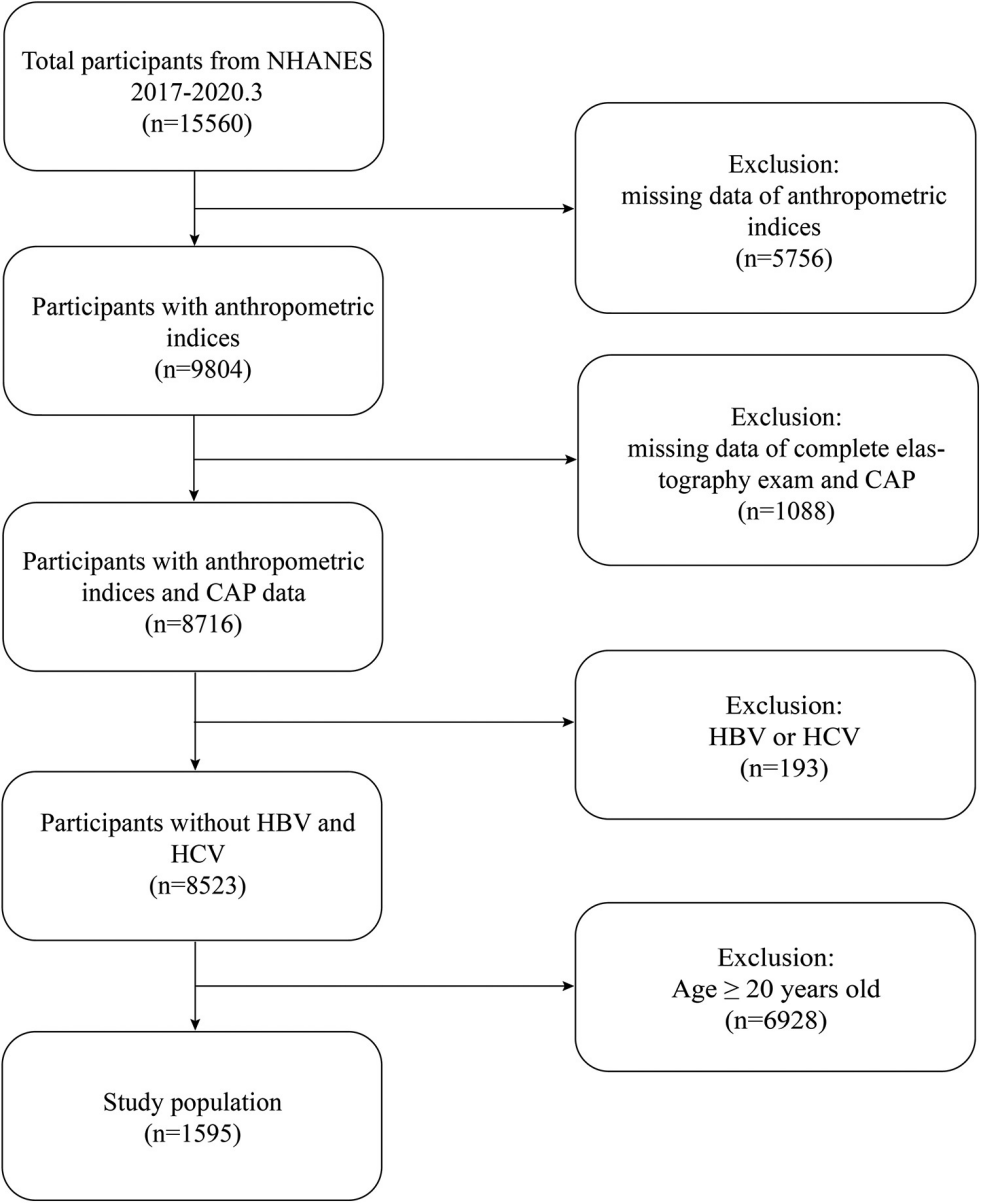
Flowchart for participant selection.

The study population was drawn from NHANES 2017–2020, focusing on participants aged 12–19 years with complete anthropometric measurements and Controlled Attenuation Parameter (CAP) data. Initially, 15,560 individuals were enrolled. After exclusions due to missing anthropometric data (5,756 participants), non-completion of liver elastography or missing CAP values (1,088 participants), as well as exclusions of individuals aged 20 years or older and those with hepatitis B or C infections (6,928 participants), a final cohort of 1,595 adolescents remained for analysis (Figure 1).

### 2.2 Definition of anthropometric indices and their measurements

According to the Protocols and Procedures documents available on the CDC's NHANES website, anthropometric measurements were obtained at the Mobile Examination Centers (MEC), where trained professionals followed standardized protocols and used validated instruments to collect data, including height, weight, waist circumference (WC), and hip circumference (HC). The nine anthropometric indices were derived using established formulas, as referenced in previous studies (15–17):

$$\mathrm{BMI}=\frac{\mathrm{weight}\;(\mathrm{kg})}{\mathrm{heigh}t^{2}\;(m)}$$

$$\mathrm{ABSI}=\;\frac{\mathrm{WC}(\mathrm{cm})}{{\left(\mathrm{BMI}\right)}^{2/3}(\mathrm{kg}/m^{2})\times \mathrm{heigh}t^{1/2}\;(m)}$$

$$\mathrm{AVI}=\frac{2\times WC^{2}\;(\mathrm{cm})+0.7\times {\left(\mathrm{WC}\;(\mathrm{cm})-\mathrm{HC}\;(\mathrm{cm})\right)}^{2}}{1000}$$

$$\mathrm{BAI}=\frac{\mathrm{HC}\;(\mathrm{cm})}{\mathrm{heigh}t^{1.5\;}(m)}-18$$

$$\mathrm{BRI}=364.2-365.5\times \sqrt{1-{\left(\frac{\mathrm{WC}\;(m)}{\pi \times \mathrm{height}\;(m)}\right)}^{2}}$$

$$\mathrm{CI}=\frac{\mathrm{WC}\;(m)}{0.109\times \sqrt{\frac{\mathrm{weight}\;(\mathrm{kg})}{\mathrm{height}\;(m)}}}$$

$$\mathrm{WHR}=\frac{\mathrm{WC}\;(\mathrm{cm})}{\mathrm{HC}\;(\mathrm{cm})}$$

$$\mathrm{WHtR}=\frac{\mathrm{WC}\;(\mathrm{cm})}{\mathrm{height}\;(\mathrm{cm})}$$

$$\mathrm{WWI}=\frac{\mathrm{WC}\;(\mathrm{cm})}{\sqrt{\mathrm{weight}\;(\mathrm{kg})}}$$

ABSI, A body shape index; AVI, abdominal volume index; BAI, body adiposity index; BMI, body mass index; BRI, body roundness index; CI, conicity index; HC, hip circumference; WC, waist circumference; WHR, waist-hip ratio; WHtR, waist-to-height ratio; WWI, weight-adjusted waist index.

Our study included nine anthropometric indices as exposure variables.

### 2.3 Hepatic steatosis evaluation

Hepatic steatosis was assessed using FibroScan® technology, a non-invasive method that utilizes ultrasound to quantify liver fat. The result is the controlled attenuation parameter (CAP), a numerical indicator of hepatic steatosis. Hepatic fat infiltration was categorized histologically into four classes: S0 (no or minimal fat, <5% hepatocytes with fat); S1 (mild fat infiltration, 5%–33% hepatocytes with fat); S2 (moderate fat infiltration, 33%–66% hepatocytes with fat); and S3 (severe fat infiltration, >66% hepatocytes with fat) (18). Based on previous studies, CAP values are classified as follows: S0 (CAP < 248 dB/m), S1 (248 ≤ CAP < 268 dB/m), S2 (268 ≤ CAP < 280 dB/m), and S3 (CAP ≥ 280 dB/m) (19). Hepatic steatosis was defined by a CAP value of 248 dB/m or higher.

### 2.4 Covariates

Several covariates were considered in this study, including age, sex, race, poverty income ratio (PIR), smoking status, and various biochemical markers, such as alanine aminotransferase (ALT), aspartate aminotransferase (AST), alkaline phosphatase (ALP), high-density lipoprotein cholesterol (HDL-C), and high-sensitivity C-reactive protein (hs-CRP). The PIR was used to measure socioeconomic status, with values of PIR < 1.3 indicating economic disadvantage and PIR ≥ 1.3 indicating economic stability (20). Smoking status was categorized based on serum cotinine levels: non-exposed (≤0.05 ng/ml), passive exposure (0.05–2.99 ng/ml), and active exposure (>2.99 ng/ml) (20). Missing data were imputed using a random forest model. Continuous variables included age, ALT, AST, ALP, HDL-C, and hs-CRP, while other variables were categorized. Detailed measurement methodologies are publicly available on the CDC's NHANES website.

### 2.5 Statistical analysis

Statistical analysis was performed using Python (version 3.10), R software (version 4.2.0), and EmpowerStats (version 6.0), following NHANES protocols for the computation and application of sample weights. Continuous and categorical variables were presented using weighted averages and 95% confidence intervals (CIs). Anthropometric indices were normalized using *Z*-scores, calculated as $\left(Z\text{-}\mathrm{Score}=\frac{\mathrm{index}-\mathrm{inde}x_{\mathrm{mean}}}{\mathrm{inde}x_{\mathrm{sd}}}\right)$.

Multivariate logistic regression analyses were conducted using three models: Model 1 (no adjustments), Model 2 (adjusted for age, sex, and ethnicity), and Model 3 (adjusted for a comprehensive set of covariates). The relationship between anthropometric indices and hepatic steatosis was further explored using smooth curve fitting techniques. The area under the receiver operating characteristic (ROC) curve (AUC) was used to assess the predictive accuracy of each anthropometric index for hepatic steatosis. Stratified multivariate logistic regression models were applied to examine how the association between AVI and hepatic steatosis varied across different demographic subgroups, including age, sex, race, income, and smoking status. The optimal cutoff value for prediction was determined using the Youden index derived from ROC curve analysis. A simple Python program was developed to alert users when their AVI exceeds a specified threshold, indicating an increased risk of hepatic steatosis. Statistical significance was determined for *P*-values below 0.05.

## 3 Results

### 3.1 Demographic and baseline attributes

Table 1 presents the demographic and baseline characteristics of the adolescents in the NHANES cohort, including detailed information on hepatic steatosis and various anthropometric measures.

**Table 1:** Baseline characteristics of participants, weighted.

Characteristic	Absent/Normal (S0)	Mild (S1)	Moderate (S2)	Severe (S3)	*P*-value
	CAP < 248	248 ≤ CAP < 268	268 ≤ CAP < 280	CAP ≥ 280	
	*N* = 1,160	*N* = 133	*N* = 81	*N* = 221	
Age, years	15.32 (15.13, 15.51)	15.17 (14.79, 15.56)	15.57 (15.13, 16.01)	15.92 (15.57, 16.26)	0.0725
Sex, *N* (%)					0.3558
Male	51.26 (45.08, 57.39)	52.02 (42.40, 61.48)	57.91 (41.48, 72.76)	59.33 (50.68, 67.44)	
Female	48.74 (42.61, 54.92)	47.98 (38.52, 57.60)	42.09 (27.24, 58.52)	40.67 (32.56, 49.32)	
Race, *N* (%)					<0.0001
Mexican American	13.20 (9.86, 17.46)	21.10 (12.22, 33.93)	16.83 (7.35, 34.06)	29.95 (19.61, 42.83)	
Other Hispanic	8.24 (6.15, 10.96)	13.46 (7.59, 22.77)	6.73 (2.85, 15.06)	7.00 (3.69, 12.90)	
Non-Hispanic White	54.35 (48.20, 60.38)	47.45 (36.76, 58.38)	57.96 (40.93, 73.28)	40.96 (28.12, 55.17)	
Non-Hispanic Black	13.35 (10.14, 17.38)	8.33 (4.60, 14.64)	12.34 (4.77, 28.36)	11.63 (6.78, 19.22)	
Other Races—including multi-racial	10.85 (8.78, 13.33)	9.66 (5.42, 16.63)	6.15 (2.45, 14.57)	10.46 (7.27, 14.82)	
PIR, *N* (%)					0.0103
<1.3	26.62 (22.74, 30.89)	29.62 (21.01, 39.97)	33.36 (23.65, 44.71)	39.32 (28.86, 50.86)	
≥1.3	73.38 (69.11, 77.26)	70.38 (60.03, 78.99)	66.64 (55.29, 76.35)	60.68 (49.14, 71.14)	
Smoking, *N* (%)					0.2561
No smoke exposure	55.77 (50.42, 60.99)	48.07 (32.88, 63.63)	44.54 (28.04, 62.33)	51.61 (41.71, 61.39)	
Passive smoker	28.05 (24.70, 31.66)	32.77 (23.86, 43.11)	43.29 (29.91, 57.71)	36.19 (27.50, 45.89)	
Active smoker	16.18 (13.46, 19.33)	19.16 (9.39, 35.15)	12.18 (5.50, 24.83)	12.20 (7.48, 19.27)	
ALT	15.74 (14.41, 17.08)	16.90 (15.83, 17.97)	18.31 (16.43, 20.19)	27.48 (24.61, 30.36)	<0.0001
AST	20.05 (19.31, 20.79)	19.67 (18.66, 20.67)	19.07 (18.06, 20.09)	22.72 (21.34, 24.11)	0.0015
ALP	148.46 (139.28, 157.63)	154.05 (130.90, 177.20)	155.90 (131.62, 180.19)	135.20 (122.34, 148.06)	0.1836
HDL-C	52.62 (51.41, 53.82)	48.51 (47.19, 49.84)	48.89 (46.57, 51.21)	45.58 (44.04, 47.12)	<0.0001
hsCRP	1.89 (1.52, 2.25)	1.95 (1.33, 2.58)	2.57 (2.02, 3.11)	3.20 (2.82, 3.58)	0.0035
BMI	22.37 (21.97, 22.77)	26.53 (25.30, 27.76)	28.67 (27.17, 30.16)	32.67 (31.53, 33.80)	<0.0001
ABSI	7.64 (7.61, 7.67)	7.79 (7.67, 7.90)	7.83 (7.72, 7.93)	7.85 (7.81, 7.89)	<0.0001
AVI	12.50 (12.16, 12.85)	16.33 (15.01, 17.64)	18.50 (17.15, 19.85)	21.97 (21.09, 22.84)	<0.0001
BAI	26.08 (25.67, 26.48)	29.63 (28.44, 30.81)	30.29 (28.92, 31.65)	33.57 (32.37, 34.78)	<0.0001
BRI	2.90 (2.78, 3.01)	4.22 (3.77, 4.67)	4.74 (4.33, 5.14)	5.95 (5.64, 6.27)	<0.0001
CI	1.17 (1.17, 1.18)	1.23 (1.21, 1.25)	1.25 (1.23, 1.27)	1.28 (1.28, 1.29)	<0.0001
WHR	0.83 (0.82, 0.83)	0.87 (0.86, 0.89)	0.89 (0.88, 0.91)	0.92 (0.91, 0.93)	<0.0001
WHtR	0.47 (0.46, 0.48)	0.54 (0.52, 0.56)	0.56 (0.54, 0.58)	0.62 (0.60, 0.63)	<0.0001
WWI	9.96 (9.90, 10.02)	10.44 (10.23, 10.64)	10.51 (10.36, 10.66)	10.79 (10.72, 10.87)	<0.0001

Data in the table: For continuous variables: survey-weighted mean (95% CI); *P*-value was calculated using survey-weighted linear regression (svyglm) For categorical variables: survey-weighted percentage (95% CI), *P*-value was calculated using survey-weighted Chi-square test (svytable). ABSI, A body shape index; AVI, abdominal volume index; ALP, alkaline phosphatase; ALT, alanine aminotransferase; AST, aspartate aminotransferase; BAI, body adiposity index; BMI, body mass index; BRI, body roundness index; CI, conicity index; HDL-C, direct high-density lipoprotein cholesterol; hsCRP, high-sensitivity C-reactive protein; PIR, poverty income ratio; WHR, waist-hip ratio; WHtR, waist-to-height ratio; WWI, weight-adjusted waist index.

The analysis involved a total of 1,595 adolescents, who were categorized into quartiles based on the degree of liver fat accumulation: S0 (1,160 participants), S1 (133 participants), S2 (81 participants), and S3 (221 participants). The average age of the participants was 15.43 years, with a standard deviation of 2.24 years. Males accounted for 53.23% of the cohort (849 individuals), while females made up 46.77% (746 individuals). Generally, anthropometric measurements associated with hepatic steatosis were higher than those in the S0 category. Severe steatosis (S3) was more prevalent among non-Hispanic Whites and adolescents with elevated levels of ALT, AST, and hs-CRP, lower levels of HDL-C, and those from more affluent backgrounds than among those in the S0 category.

### 3.2 Correlation between anthropometric measures and hepatic steatosis

**Figure 2:**
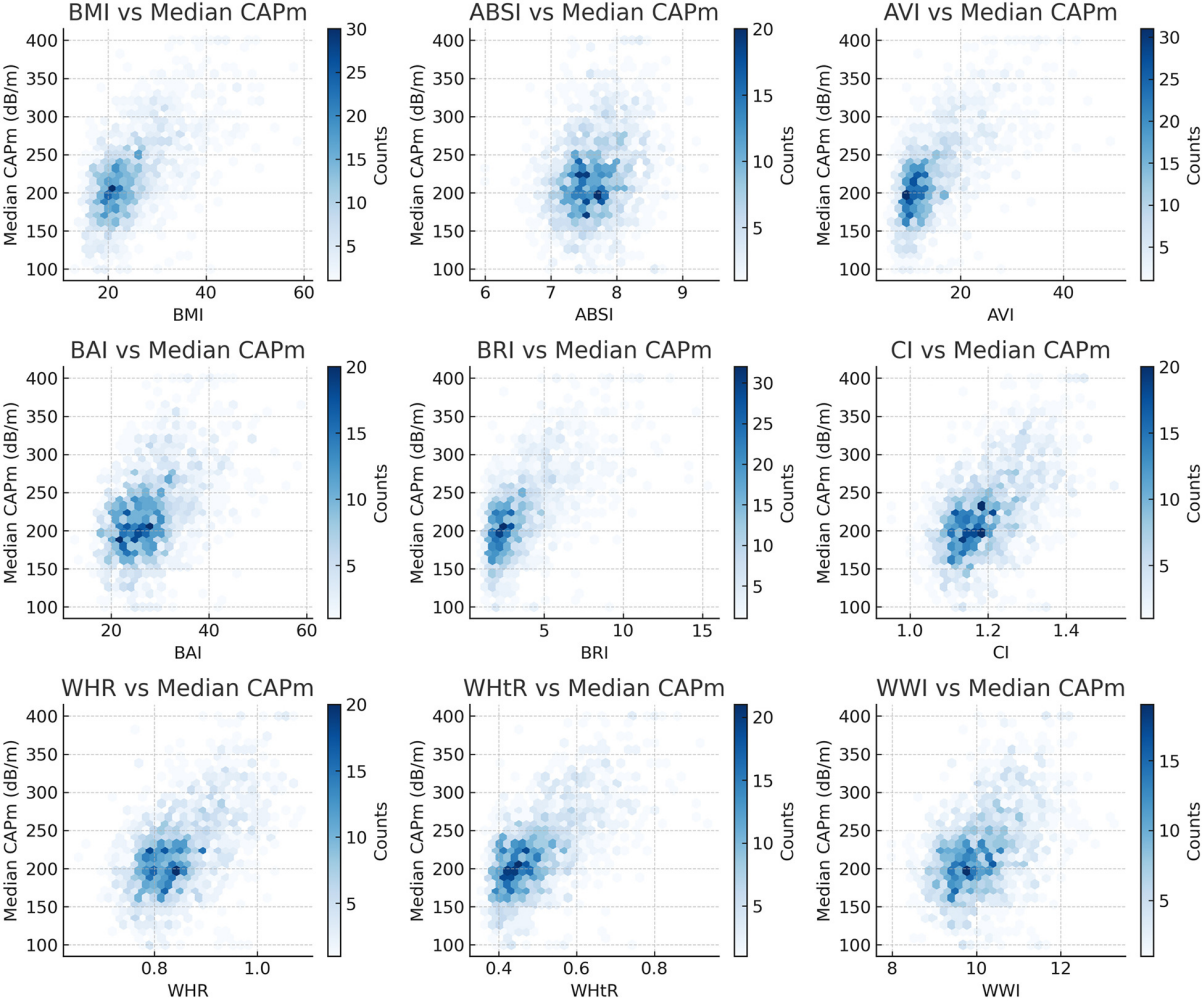
Relationship between anthropometric indices and median CAPm.

**Figure 3:**
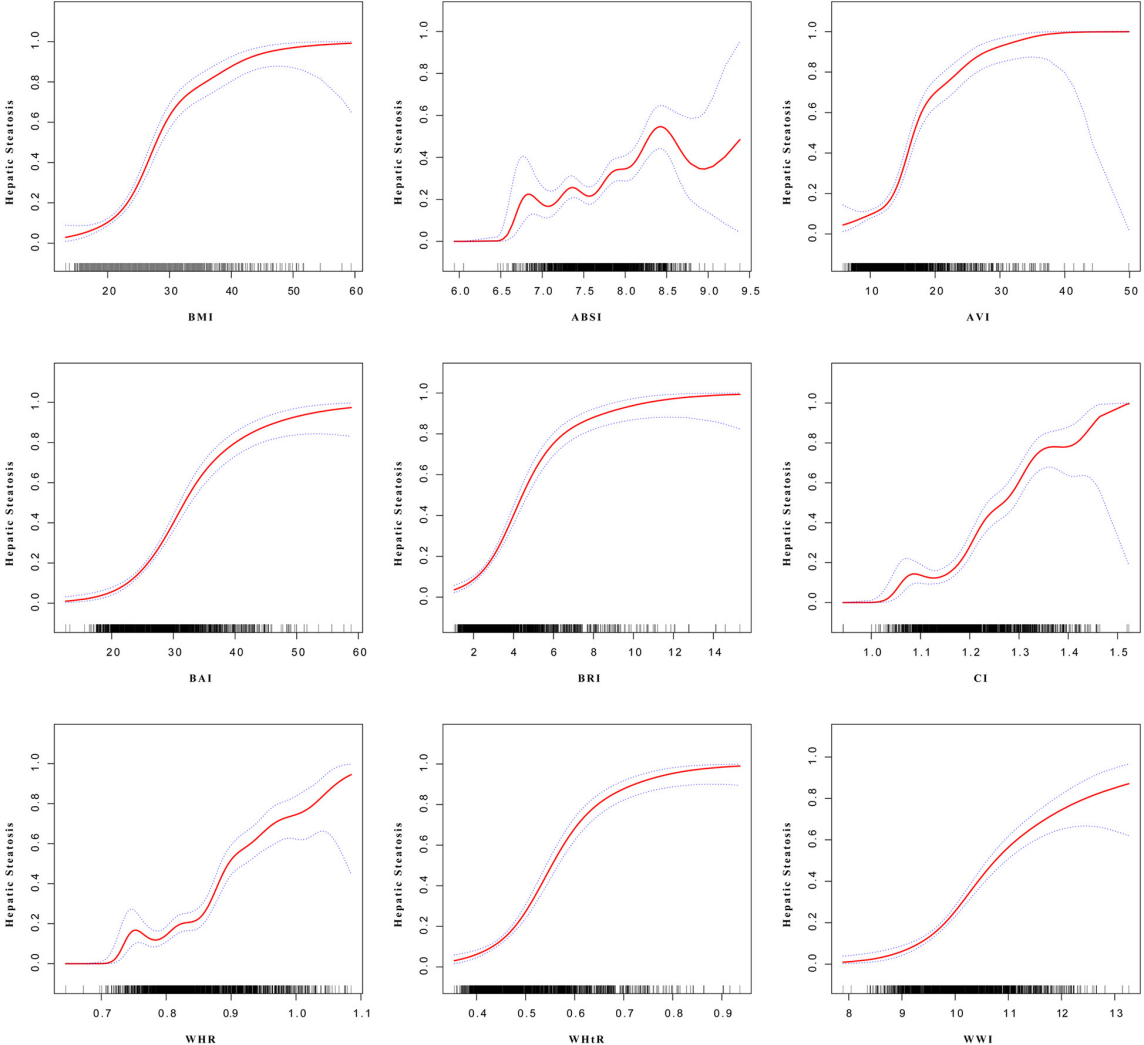
Nonlinear relationship between anthropometric indicators and hepatic steatosis. The red solid line indicates a smooth curve fit between variables. The blue bands indicate the 95% confidence interval from the fit.

A comprehensive logistic regression analysis was conducted to examine the relationship between hepatic steatosis and various anthropometric indices. Following *z*-score standardization, all evaluated indices showed a positive association with hepatic steatosis, as summarized in Table 2. In the unadjusted model (Model 1), AVI presented the highest odds ratio (OR) for every 1 standard deviation increase (OR: 4.88; 95% CI: 3.73–6.39, *P* < 0.0001), outperforming all other anthropometric indicators. After adjusting for age, sex, ethnicity, PIR, nicotine use status, and serum levels of ALT, AST, ALP, HDL-C, and hs-CRP (Model 3), all indices remained significantly associated with hepatic steatosis. Specifically, the odds ratios for the anthropometric measures in Model 3 were as follows: BMI (OR: 4.71; 95% CI: 3.70–5.99, *P* < 0.0001), ABSI (OR: 1.51; 95% CI: 1.24–1.84, *P* = 0.0023), AVI (OR: 5.08; 95% CI: 3.86–6.68, *P* < 0.0001), BAI (OR: 3.79; 95% CI: 3.07–4.68, *P* < 0.0001), BRI (OR: 4.73; 95% CI: 3.78–5.94, *P* < 0.0001), CI (OR: 3.15; 95% CI: 2.67–3.72, *P* < 0.0001), WHR (OR: 3.01; 95% CI: 2.52–3.60, *P* < 0.0001), WHtR (OR: 4.62; 95% CI: 3.76–5.67, *P* < 0.0001), and WWI (OR: 2.73; 95% CI: 2.35–3.17, *P* < 0.0001), with AVI maintaining the highest OR. The correlation between each anthropometric index and hepatic steatosis as indicated by CAP is depicted in Figure 2. Furthermore, the results from smooth curve fitting revealed a nonlinear relationship between the anthropometric indices and the incidence of hepatic steatosis in adolescents, as shown in Figure 3.

**Table 2:** Multivariate logistic regression analysis of anthropometric indices and hepatic steatosis.

Hepatic steatosis	Model 1		Model 2		Model 3	
	OR (95% CI)	*P*-value	OR (95% CI)	*P*-value	OR (95% CI)	*P*-value
BMI *Z*-score	4.41 (3.52, 5.53)	<0.0001	4.91 (3.80, 6.36)	<0.0001	4.71 (3.70, 5.99)	<0.0001
ABSI *Z*-score	1.62 (1.41, 1.85)	<0.0001	1.67 (1.46, 1.91)	<0.0001	1.51 (1.24, 1.84)	0.0023
AVI *Z*-score	4.88 (3.73, 6.39)	<0.0001	5.17 (3.86, 6.94)	<0.0001	5.08 (3.86, 6.68)	<0.0001
BAI *Z*-score	2.92 (2.53, 3.38)	<0.0001	4.27 (3.43, 5.30)	<0.0001	3.79 (3.07, 4.68)	<0.0001
BRI *Z*-score	4.68 (3.69, 5.95)	<0.0001	4.90 (3.80, 6.32)	<0.0001	4.73 (3.78, 5.94)	<0.0001
CI *Z*-score	3.53 (2.87, 4.33)	<0.0001	3.50 (2.87, 4.27)	<0.0001	3.15 (2.67, 3.72)	<0.0001
WHR *Z*-score	3.37 (2.75, 4.11)	<0.0001	3.37 (2.75, 4.14)	<0.0001	3.01 (2.52, 3.60)	<0.0001
WHtR *Z*-score	4.53 (3.64, 5.65)	<0.0001	4.76 (3.76, 6.02)	<0.0001	4.62 (3.76, 5.67)	<0.0001
WWI *Z*-score	2.76 (2.35, 3.24)	<0.0001	3.08 (2.61, 3.62)	<0.0001	2.73 (2.35, 3.17)	<0.0001

Model 1: unadjusted model. Model 2: Adjusted for age, sex, race. Model 3: Adjusted for age, sex, race, PIR, smoking status, ALT, AST, ALP, HDL-C, and hsCRP. ABSI, A body shape index; AVI, abdominal volume index; ALP, alkaline phosphatase; ALT, alanine aminotransferase; AST, aspartate aminotransferase; BAI, body adiposity index; BMI, body mass index; BRI, body roundness index; CI, conicity index; HDL-C, direct high-density lipoprotein cholesterol; hsCRP, high-sensitivity C-reactive protein; PIR, poverty income ratio; WHR, waist-hip ratio; WHtR, waist-to-height ratio; WWI, weight-adjusted waist index.

### 3.3 ROC curves and AUC for indices in detecting hepatic steatosis

**Figure 4:**
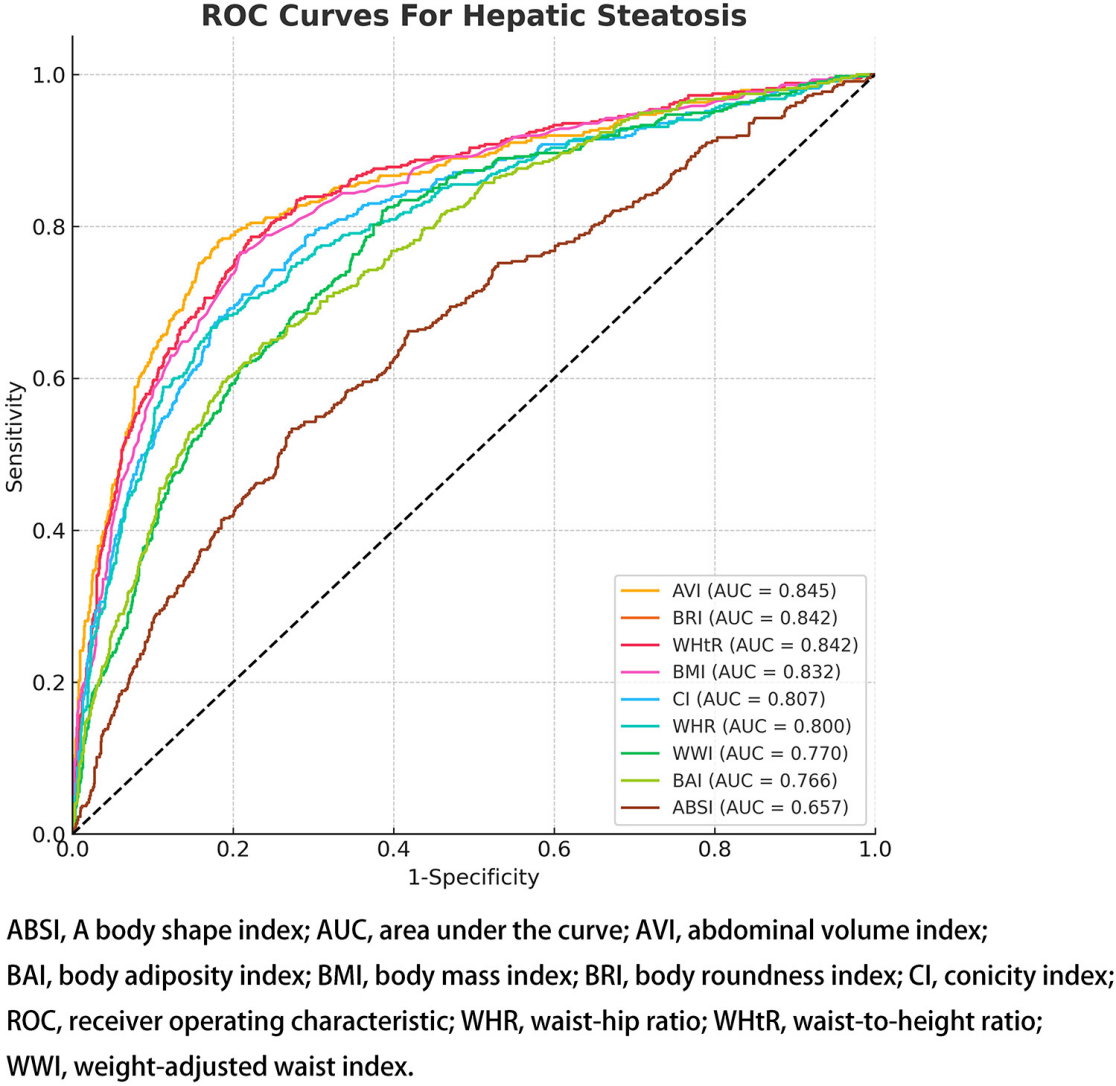
ROC curves representing anthropometric indices used for differentiating hepatic steatosis.

To evaluate the diagnostic performance of the different anthropometric indicators in identifying adolescents with hepatic steatosis, we generated ROC curves and calculated the AUC), as presented in Table 3 and Figure 4. Among the nine indices, AVI demonstrated the highest predictive accuracy (AUC = 0.8454, 95% CI: 0.8221–0.8687, best threshold: 14.9992). Other indices such as BRI, WHtR, BMI, CI, and WHR also showed robust AUC values, indicating their strong diagnostic potential.

**Table 3:** ROC analysis for continuous predictors.

Test	Best threshold	ROC (AUC)	95% CI low	95% CI upp	Specificity	Sensitivity
BMI	25.4500	0.8320	0.8086	0.8554	0.7914	0.7655
ABSI	7.8160	0.6572	0.6266	0.6878	0.7267	0.5333
AVI	14.9992	0.8454	0.8221	0.8687	0.8172	0.7816
BAI	29.4317	0.7656	0.7394	0.7917	0.7629	0.6460
BRI	3.5725	0.8417	0.8189	0.8645	0.7776	0.7862
CI	1.2180	0.8069	0.7816	0.8321	0.7879	0.7103
WHR	0.8775	0.7997	0.7739	0.8255	0.8276	0.6667
WHtR	0.5113	0.8417	0.8189	0.8645	0.7776	0.7862
WWI	10.0479	0.7703	0.7444	0.7962	0.6138	0.8207

ABSI, A body shape index; AUC, area under the curve; AVI, abdominal volume index; BAI, body adiposity index; BMI, body mass index; BRI, body roundness index; CI, conicity index; ROC, receiver operating characteristic; WHR, waist-hip ratio; WHtR, waist-to-height ratio; WWI, weight-adjusted waist index.

### 3.4 Subgroup analysis

Given the superior predictive accuracy of AVI among the indices, a subgroup analysis was conducted to explore how the association between AVI and hepatic steatosis varied across different demographic groups. The analysis revealed a consistent positive correlation between AVI and hepatic steatosis across all subgroups. Interaction tests indicated that race significantly influenced the relationship between AVI and hepatic steatosis (*P* for interaction < 0.05). Specifically, adolescents from multi-racial and other racial backgrounds exhibited a stronger association between AVI and hepatic steatosis than Mexican Americans, other Hispanics, non-Hispanic Whites, and non-Hispanic Blacks. However, age, sex, PIR, and smoking status did not significantly alter this relationship (*P* for interaction > 0.05; Table 4).

**Table 4:** Subgroup analysis of the association between AVI and hepatic steatosis.

Subgroup	OR (95% CI)	*P* for interaction
Age		0.1904
12–15	1.28 (1.20, 1.36)	
16–19	1.35 (1.28, 1.43)	
Sex		0.2539
Male	1.35 (1.25, 1.45)	
Female	1.28 (1.20, 1.35)	
Race		<0.0001
Mexican American	1.44 (1.22, 1.69)	
Other Hispanic	1.38 (1.21, 1.57)	
Non-Hispanic White	1.30 (1.22, 1.39)	
Non-Hispanic Black	1.18 (1.12, 1.26)	
Other races—including multi-racial	1.54 (1.39, 1.72)	
PIR		0.0781
<1.3	1.40 (1.27, 1.54)	
≥1.3	1.28 (1.22, 1.34)	
Smoking		0.0981
No smoke exposure	1.39 (1.30, 1.48)	
Passive smoker	1.26 (1.17, 1.36)	
Active smoker	1.28 (1.17, 1.41)	

AVI, abdominal volume index; CI, confidence interval; OR, odds ratio; PIR, poverty income ratio.

### 3.5 Predictive program design

A Python-based tool was developed to assist in calculating anthropometric indices associated with hepatic steatosis in adolescents. The program uses measurements such as height, weight, waist circumference, and hip circumference to compute AVI. It compares the calculated AVI to a threshold value of 14.9992, previously identified as optimal for predicting hepatic steatosis risk. If AVI meets or exceeds this threshold, the tool alerts the user to a potentially elevated risk of hepatic steatosis, recommending a consultation with a healthcare professional. Conversely, if AVI is below the threshold, the user is informed that their risk is not immediately significant, although maintaining a healthy lifestyle is still encouraged (Supplementary Material).

## 4 Discussion

This study, one of the first of its kind, investigated the relationship between hepatic steatosis and a range of anthropometric indices in a cohort of American adolescents, providing valuable insights into the broader demographic. Utilizing pre-pandemic NHANES data from 2017 to March 2020, we examined how nine different anthropometric indices correlate with hepatic steatosis in this age group. Our findings show that, after accounting for various factors, there is a positive association between these indices and hepatic steatosis. Notably, AVI displayed the strongest discriminative power for detecting hepatic steatosis in adolescents, with an AUC of 0.8454 (95% CI: 0.8221–0.8687), indicating excellent sensitivity and specificity. In addition, indices such as BRI, WHtR, BMI, CI, and WHR also exhibited elevated AUC values, suggesting their potential utility in the preliminary identification of hepatic steatosis among adolescents.

The complex relationship between obesity and hepatic steatosis has garnered significant scientific attention. Obesity-induced insulin resistance plays a pivotal role in the development of hepatic steatosis by triggering lipolysis in adipose cells, which releases free fatty acids (FFAs) into the bloodstream. These FFAs are then taken up by the liver, where they are converted into triglycerides, disrupting lipid metabolism and leading to hepatic fat accumulation (21). Additionally, obesity promotes chronic inflammation, with adipose tissue acting as a source of pro-inflammatory cytokines such as TNF-α, IL-6, and IL-1β, which exacerbate lipid deposition in the liver (22). The elevated fatty acid levels in the liver can induce oxidative stress and mitochondrial dysfunction, further damaging hepatocytes and accelerating the progression of steatosis (23). Recent studies have highlighted the role of impaired hepatic fatty acid oxidation (FAO) and mitochondrial turnover in the severity of NAFLD in obese individuals (24). Moreover, gut microbiome imbalances, or dysbiosis, may contribute to the worsening of obesity-related hepatic steatosis by affecting energy metabolism and inflammatory responses (25, 26). Research by Sergio et al. has shown that histidine supplementation can reduce Proteobacteria abundance in NAFLD models, improving hepatic steatosis, inflammation, and insulin resistance (27). Genetic and epigenetic factors also influence liver lipid metabolism, increasing the risk of steatosis. These interconnected mechanisms help explain the rising prevalence of hepatic steatosis in obese adolescents, which, if left untreated, may progress to non-alcoholic steatohepatitis, liver fibrosis, and, in severe cases, cirrhosis or liver cancer.

Anthropometric indices are critical tools for assessing obesity and its associated health risks. While the widely used BMI is a general indicator of obesity, it is limited in its ability to predict metabolic risks linked to visceral fat. This is because BMI does not distinguish between adipose and muscular tissues, nor does it account for fat distribution patterns (28–30). In contrast, indices such as AVI and BRI are more accurate representations of abdominal and visceral fat accumulation (31, 32) and are more strongly correlated with obesity-related diseases, including cardiovascular disease, diabetes, and NAFLD.

Hepatic steatosis is closely associated with several metabolic disorders, including insulin resistance, lipid metabolism dysfunction, and chronic inflammation (33–35). The accumulation of visceral fat plays a significant role in the development of hepatic steatosis in adolescents, and anthropometric indices serve as indirect measures of the risk for liver fat accumulation. In our study, nearly all the anthropometric measures demonstrated a positive correlation with hepatic steatosis, highlighting their utility as predictive tools. However, AVI stood out as the most accurate predictor, reflecting its higher sensitivity in detecting increases in visceral fat.

AVI, as a specific marker for abdominal fat accumulation, provides a more precise assessment of the relationship between visceral fat and metabolic diseases (36). Our results indicate that AVI has a stronger association with hepatic steatosis than other anthropometric indices, likely because it more effectively reflects visceral fat accumulation, making it a superior predictor of hepatic steatosis risk. Recent studies in adolescents have also shown that cardiovascular risk indices are closely related not only to hepatic steatosis but also to hyperinsulinemia and insulin resistance (37). Insulin resistance promotes lipolysis in adipose tissue, increasing the transport of FFAs to the liver, which in turn enhances hepatic triglyceride synthesis and accumulation. Moreover, chronic low-grade inflammation and oxidative stress may jointly contribute to both insulin resistance and hepatic steatosis, with visceral fat accumulation (as reflected by AVI and similar indices) serving as a central factor in this process. Furthermore, even when accounting for factors such as sex, age, and ethnicity, AVI continued to show a significant correlation with hepatic steatosis, reinforcing its potential as an independent predictor.

Our subgroup analysis revealed notable racial differences in the relationship between AVI and hepatic steatosis. Specifically, the association between AVI and hepatic steatosis was stronger in adolescents of other races, including multi-racial individuals, than in Mexican Americans, other Hispanics, non-Hispanic Whites, and non-Hispanic Blacks. These disparities may be due to variations in genetics, lifestyle, and environmental factors affecting liver fat metabolism, and suggest that future research should investigate the mechanisms underlying these racial differences. While factors such as age, sex, PIR, and smoking status did not significantly alter the association between AVI and hepatic steatosis, these findings emphasize the need for personalized approaches in managing liver health.

The clinical implications of this study are significant. First, AVI, as a convenient and non-invasive measure, can be used for early screening of liver health in adolescents, particularly in primary care settings with limited resources. This would allow for timely intervention in high-risk populations. Second, as AVI gains wider clinical adoption, future research should focus on refining its threshold values to enhance its applicability across different racial and demographic groups. Longitudinal studies are also needed to assess AVI's predictive value over time in the progression of hepatic steatosis and to explore its potential in combination with other biomarkers to develop a more comprehensive system for liver health assessment.

Despite providing strong support for the use of anthropometric indices, particularly AVI, as screening tools for hepatic steatosis in adolescents, this study has several limitations. First, our assessment of hepatic steatosis relied on FibroScan® CAP values, which, although widely used in clinical practice, have some limitations in accuracy when compared to liver biopsy (38). Second, while the NHANES dataset includes a racially diverse adolescent population from the US, the generalizability of these findings to other geographic regions or ethnic groups requires further investigation. It is also important to note that our study population was limited to adolescents aged 12–19 years, and thus the results may not be directly applicable to other age groups. In addition, due to limitations in the available data, we were unable to include several important factors that may contribute to hepatic steatosis, such as alcohol consumption, drug-induced liver injury (e.g., acetaminophen-related steatosis) (39), and certain underlying conditions such as celiac disease (40). Finally, as this is a cross-sectional study, causal relationships between anthropometric indices and hepatic steatosis cannot be established. Longitudinal studies are needed to further validate the long-term diagnostic utility of AVI.

## 5 Conclusion

In conclusion, AVI demonstrates considerable diagnostic potential for predicting hepatic steatosis in adolescents, providing valuable evidence to support its use in early screening and prevention. With the global rise in adolescent obesity, AVI—being a simple and effective anthropometric index—holds significant promise for broader application. Its ease of measurement makes it particularly suitable for implementation not only in primary care settings but also in school-based health programs.

Schools can incorporate AVI assessments into routine physical examinations to establish student health profiles and offer basic, personalized health advice. For students with abnormal AVI values, school physicians can conduct regular follow-ups to monitor the effectiveness of lifestyle interventions, such as increased physical activity and dietary modifications. In more serious cases, schools may notify parents and assist with referrals to specialized outpatient clinics at higher-level medical institutions (e.g., pediatric endocrinology) for further evaluation.

Meanwhile, parents can independently monitor their child's AVI at home using simple measurement tools or the program developed in this study, which is based on the AVI algorithm. This enables timely and user-friendly risk assessment, facilitating early detection and encouraging prompt medical consultation when necessary.

Together, these efforts contribute to a multi-tiered screening and management framework—from home and school monitoring to primary care and advanced clinical referral—thereby enhancing the translational value of AVI in real-world settings. This comprehensive approach not only supports early identification and intervention for hepatic steatosis in adolescents but also promotes greater public awareness and engagement in metabolic health.

## Data Availability

The datasets presented in this study can be found in online repositories. The names of the repository/repositories and accession number(s) can be found below: The data supporting the study's findings is readily available through the NHANES platform.
