# Supplementary material for: Nine anthropometric indices predict hepatic steatosis and assess liver health in adolescents: a population-based study
Source: Front Pediatr. 2025 Jun 9;13:1558023. doi: 10.3389/fped.2025.1558023 (PMC12183296; doi:10.3389/fped.2025.1558023)
Supplement: Supplementary file 1 [file Datasheet1.pdf]

Supplementary material

A simple Python program for predicting hepatic steatosis in users.

① Click this program.

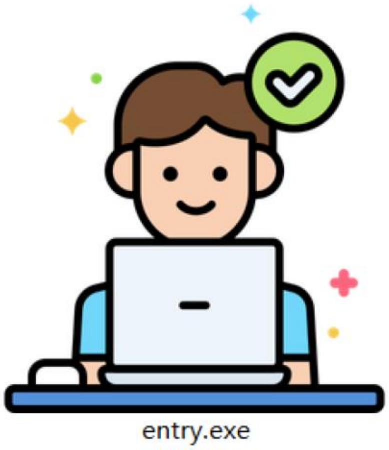

②

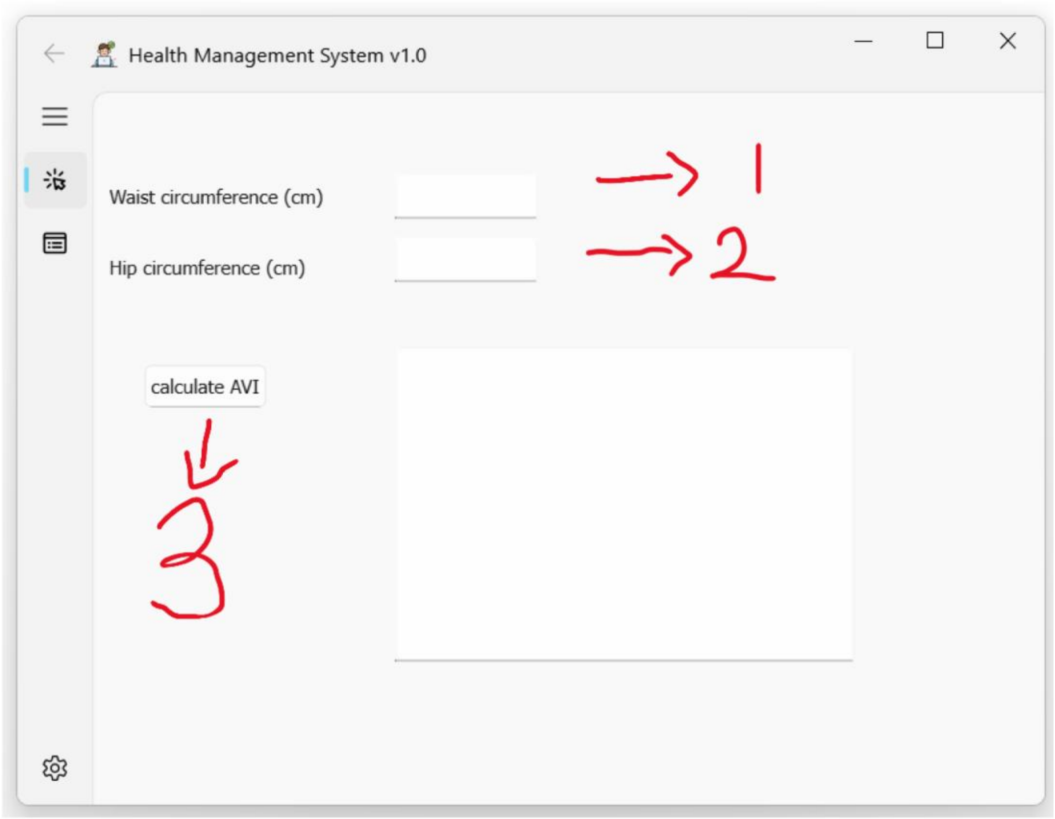

③ **Eg:**

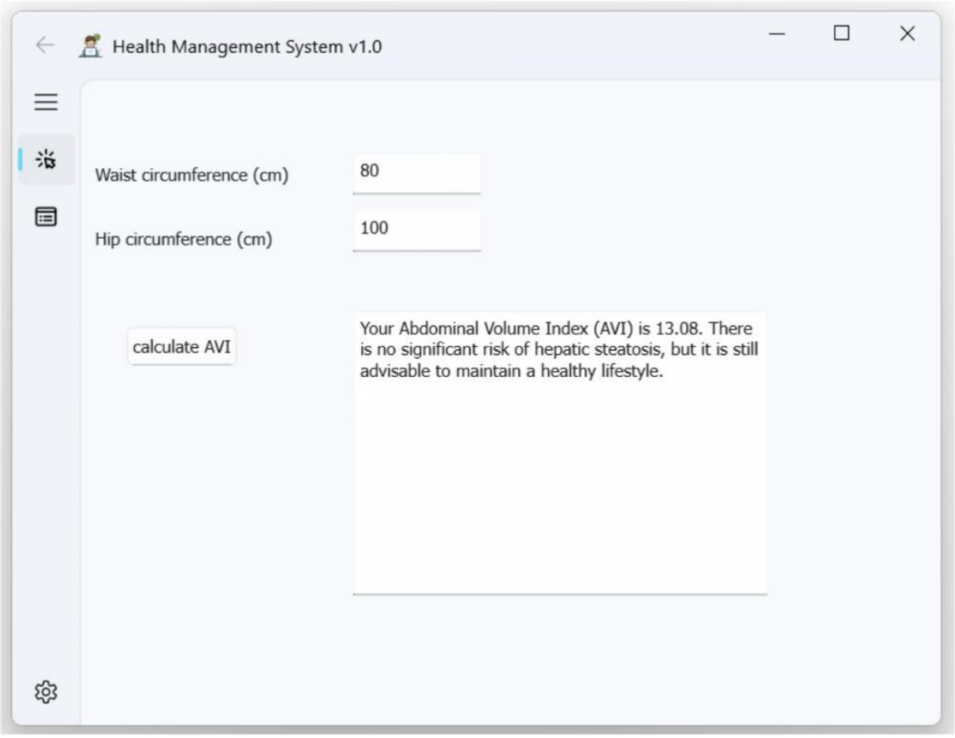
Health Management System v1.0
Waist circumference (cm)
80
Hip circumference (cm)
100
calculate AVI
Your Abdominal Volume Index (AVI) is 13.08. There is no significant risk of hepatic steatosis, but it is still advisable to maintain a healthy lifestyle.

④

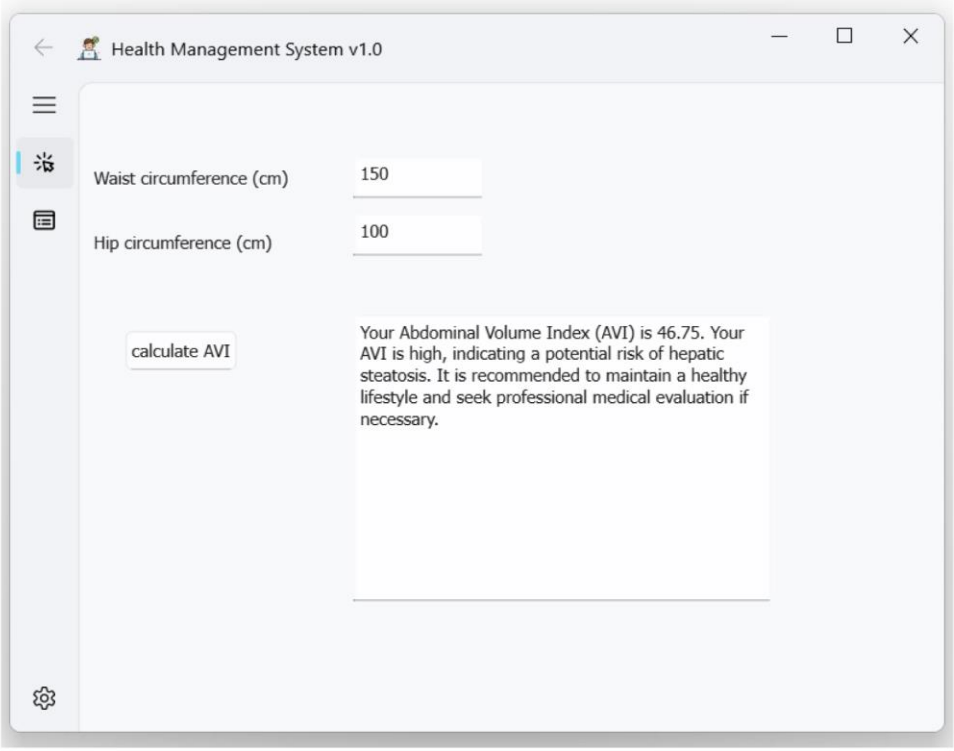
Health Management System v1.0
Waist circumference (cm)
150
Hip circumference (cm)
100
calculate AVI
Your Abdominal Volume Index (AVI) is 46.75. Your AVI is high, indicating a potential risk of hepatic steatosis. It is recommended to maintain a healthy lifestyle and seek professional medical evaluation if necessary.

⑤

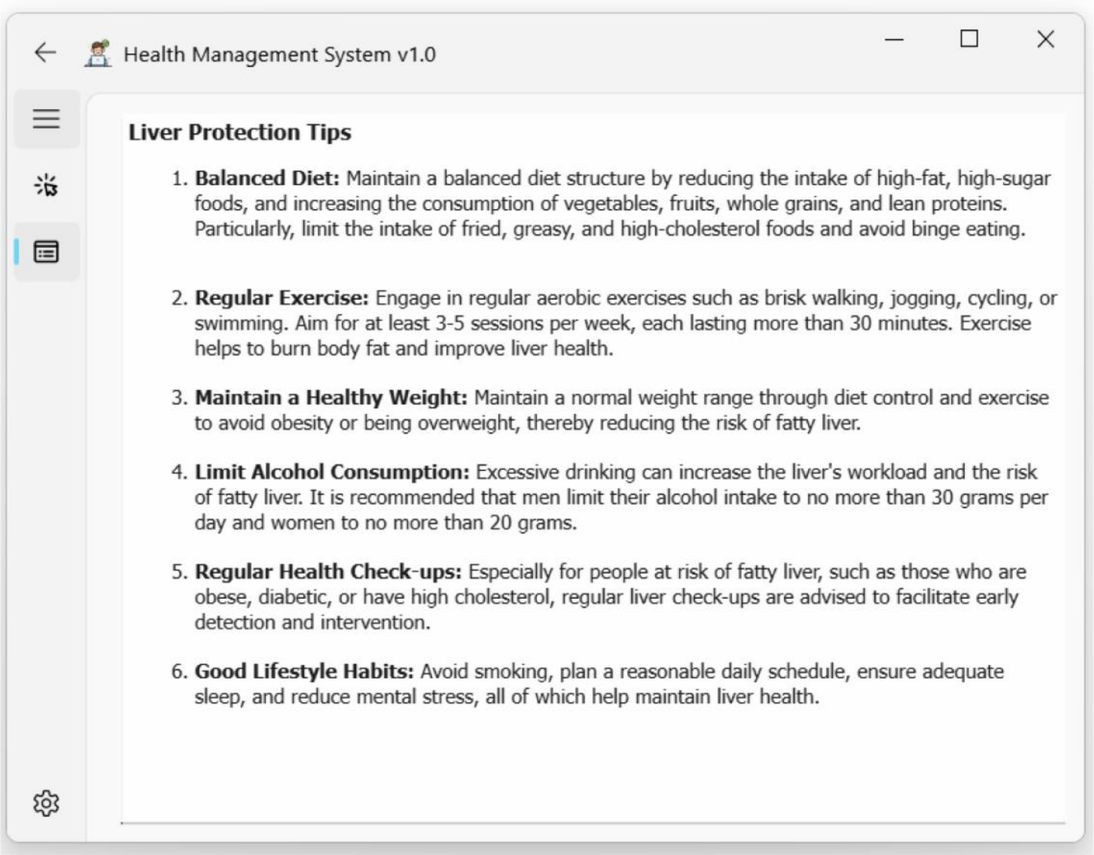

⑥ Please contact us if you want to test this program.
